# Thoughts on Respiration

**Published:** 1871-01

**Authors:** Geo. Watt


### DENTAL REGISTER.

Vol. XXV.] JANUARY, 1871. [No. 1.

### Communications.

### THOUGHTS ON RESPIRATION.

BY GEO. WATT.

Read before the Ohio State Dental Society.

The chief objects of breathing are to get oxygen into the blood, and to get oxydized matter out of it. We are far too apt to overlook the latter, or to regard it as of minor importance.

The atmosphere is a mixture of nitrogen, oxygen and carbonic acid. Other gases and vapors are ordinarily mixed with it, but are not essential to it. Nitrogen is the most abundant, while oxygen is regarded as the most important of the three. In speaking of respiration it has been common to refer to nitrogen as merely diluting the oxygen; and the results of breathing unmixed oxygen are referred to as confirmatory, inasmuch as the vital functions are thereby over-stimulated. But that this view is incorrect is evident on a slight consideration. If the nitrogen of the atmosphere were blotted out of existence, its oxygen would continue to occupy the same space that it now does, and the expansion of the chest would result in the inhalation of the same quantity of oxygen as when the nitrogen is present. But the muscles of respiration in expanding the chest would in that case be aided by only about three pounds to the square inch, instead of fifteen as with the present atmosphere.

By respiration, oxygen is conveyed to the air-cells of the lungs, and passes through the mucous membrane which lines them, to reach the blood; and carbonic acid escapes from the blood through the same membrane, and is carried away by the expiration. It is unfortunate that the terms endosmose and exosmose have been applied to this interchange of gases. If these had been reserved for use in referring to the passage of liquids through membranous tissue, our thoughts would have been clearer. The gases obey the laws of gaseous diffusion, just as they would, under the same physical conditions, out of the body. When a liquid charged with carbonic acid is placed in a cylinder, and pressure is made on it by a piece of membrane stretched like a drumhead over a hollow piston, the acid escapes through the membrane. In like manner, when the venous blood, charged with carbonic acid, reaches the lungs, and the chest is expanded by the respiratory muscles, the atmosphere, with its pressure of fifteen pounds to the inch, rushes in, extends the membrane to its utmost stretch, and yielding to the pressure, the gas passes through the membrane into the cavity of the air-cell. This is simply obedience to the law of gaseous diffusion.

The act of expiration by no means empties the air-cells. They are as full at its close as at the completion of the inspiration. The inhaled air has usually a temperature lower than that of the lungs. In reaching the equilibrium, it expands, and the pressure thus made facilitates the passage of oxygen into the blood. The oxygen is thus steadily making its ingress, while the carbonic acid passes outward mainly during the inspiration. In both there is merely obedience to the law of diffusion.

The oxygen having reached the blood, finds the red corpuscles ready to receive it, and having received it, these bodies are found of a florid color, whereas they were previously livid. The red corpuscles contain iron. It is found in no other tissue of the body. This is worthy of special note, in view of the behavior of iron and oxygen with each other. When 28 parts of iron unite with 8 of oxygen, the protoxyd of iron is formed. With this as a base, acids unite to form various salts of iron; but as a separate compound it is not permanent, being readily oxydized to a higher degree, that is till the 28 parts of iron are united with 12 parts of oxygen, when the compound is called the sesquioxyd (or peroxyd) of iron. Carbonic acid combines readily with the protoxyd of iron to form a carbonate; but with the sesquioxyd it does not unite. And further, if the carbonate of the protoxyd is exposed to oxygen, as in atmospheric air, it is decomposed, the protoxyd being converted into the sesquioxyd of iron, and the carbonic acid, unable to unite with it, escapes as a gas.

Let us start with the blood at the right ventricle of the heart. It is here livid, or venous blood. And, for the present, let us assume that the iron in the red globules is here in the form of the protoxyd and in combination with carbonic acid. The ventricle contracts, and drives the blood to the lungs. Here it meets with oxygen which changes the protoxyd to the sesquioxyd, and as the carbonic acid has no affinity for this, it resumes its gaseous form, and escapes into the air-cells, through the membrane, aided by the pressure incident to the inspiration. The blood thus changed returns to the heart, carrying sesquioxyd, instead of carbonate of iron, and is driven through the arterial system to reach the capillaries. Here the oxygen is given out by the corpuscles, the sesquioxyd is reduced to a protoxyd, carbonic acid, already formed by the oxydation of carbon, unites with it, and the blood is changed to a livid hue, and, under the name of venous blood, is carried by the veins to the right auricle, and thence passes to the right ventricle, to be driven to the lungs to unload the carbonic acid and receive a fresh supply of oxygen, as already described. With this view the change from arterial to venous blood and back again is not mysterious.

The reception of oxygen by the corpuscles, and their giving it out in the capillaries, have been accounted for, but the above does not account for all the oxygen carried by these bodies. They receive and carry more than is necessary to change their iron from a protoxyd to a sesquioxyd. For convenience, let us call this the oxygen of reserve, to distinguish it from that used in combining with the iron.

It will aid us now to consider briefly a few of the properties of oxygen, or, at least, to recognize its active and passive states. These may be better appreciated by using a more familiar element in illustration. Iron is ordinarily in its active state. In this state it acts energetically on nitric acid, depriving it of a part of its oxygen, that it may itself become oxydized. Any excess of the acid present then unites with the oxyd thus formed, the result being nitrate of iron. It is more common to speak of nitric acid acting on iron, but the above is the order of action. But if the iron be changed to its passive state, which is simply an electric change and may be effected in several ways, it may be introduced into nitric acid and be allowed to remain there, without the slightest chemical action taking place. Like iron, oxygen has its active and passive states. In a nascent condition, that is, when it is being liberated from a compound, it is always active. Electric sparks passing through it render it active, when it is usually called ozone. Much of the oxygen in the atmosphere is changed to ozone when there is great electric disturbance; and that is the reason our instruments rust at such times, in spite of our best attention. Now let it be granted that the oxygen of reserve is passive, and we can understand why its presence is not incompatible with the life of the corpuscle. Let it be active, and we can scarcely believe that the vitality of the corpuscle will be able to resist its affinity for the phosphorized fat contained therein. The variation of a half a degree in the oxydation of the iron is compatible with the life of the corpuscle; but it is probable that if the oxygen of reserve were to assert the affinity of an active state, death to the corpuscle would at once ensue.

The four elements, carbon, hydrogen, oxygen and nitrogen, are prominent constituents of living bodies. The oxygen used in building up the several tissues is probably all derived from the food, obeying the same laws of assimilation as the other elements. The oxygen introduced by respiration serves totally different purposes. By the oxydation of carbon, hydrogen, etc., heat is generated, as if these elements were burned out of the body. Not only is heat generated, but vital force is manifested as a result of similar oxydation; or, on the principle of the correlation of forces, chemical affinity is changed into vital force. Vital force is manifested in organic and animal life. Organic life is independent of the will, while voluntary motion, thought, etc., are manifestations of animal life. As a part of the oxygen received in respiration unites with the iron in the corpuscles, while in the lungs, and is given out again in the capillaries, and as this takes place with each round of the blood, asleep or awake, wholly independent of the will, it is probable that this oxygen imparts vital force for the organic or involuntary functions, while for voluntary efforts, whether muscular or mental, the supply is obtained from the oxygen of reserve. The only mystery in this is how the oxygen of reserve is rendered active, so as to assert its affinities on the proper materials and at the proper time to give the various manifestations of vital force in obedience to the will; but this is only another illustration of the power of spirit over matter, proving the truth of the Bible statement that man is created in the image of God who said "Let there be, and there was;" and after all, it is not more strange that oxygen should be rendered active by the will than by an electric spark.

Thus far respiration has been spoken of as if its results and phenomena were uniform and constant. They are so to the extent that oxygen is taken into the blood and carbonic acid is given out with each breath. But the amount of oxygen appropriated varies greatly with the circumstances; and the liberation of carbonic acid is subject to similar variation. Nor is it when the most oxygen is taken in that the most carbonic acid is given out. There is not much difference in the quantities of oxygen used by a man at work and a man idle; but the former gives off much greater amounts of carbonic acid and water than the latter. A man asleep appropriates oxygen far more rapidly than a man awake, even if engaged in brisk labor, but at the same time he gives off far less carbonic acid and water. Awake, he appropriates oxygen enough to carry on the organic functions, that is, to sesquioxydize the iron; asleep, he lays in the oxygen of reserve, to fit him for voluntary labor and thought. The object of sleep, and its importance, are thus clearly and practically apparent.

Sleep is not the only thing requisite to the accumulation of the oxygen of reserve. The assimilation of nitrogenous food seems to be necessary. At any rate, sleep is less refreshing after a free indulgence in carbonaceous food; and when it has been demonstrated that less oxygen is appropriated in such sleep, than in sleep after partaking of food rich in nitrogen, we can understand why lean meats, oysters, fish, fowls, etc., are popularly indulged in at late suppers. And if a lack of nitrogen in the food undergoing assimilation during sleep prevents the accumulation of the normal amount of oxygen of reserve, or renders the corpuscles unfit to hold it in its passive state, they will be found all the more ready to yield what they have, producing a restless irritability incident to a constitution unrefreshed by slumber. And since this discovery has been made, we are not surprised to find "vegetarians" wakeful in the hours devoted to sleep, falling asleep in the midst of conversation, purposing everything but persisting in nothing, gradually becoming more stable as their instincts or judgments cause them to indulge freely in such vegetables as are rich in nitrogen.

From the above it follows that our voluntary exertions are sustained by oxygen laid in during previous sleep; hence when our breathing is quickened by active exercise the increased quickness is not to get more oxygen into the blood, but to get carbonic acid out of it.

It is worthy of notice that, in normal breathing, the expiration occupies much less time than the inspiration. This sudden ejection seems necessary to prevent the reabsorption or rather return of a portion of the carbonic acid into the blood, hence when there is dilatation of the air-cells, spasmodic asthma, or any cause retarding the expiration, the patient suffers as if breathing an atmosphere overcharged with carbonic acid. A common, and a very serious fault in administering anæsthetics, is a neglect to permit the utmost freedom of expiration. This remark applies alike to the use of nitrous oxyd and chloroform. When the expiration is perfectly free, delirium seldom results from the use of either.

With these principles kept in view, we can readily understand the action of a number of poisonous gases when inhaled. Hydrosulphuric acid, sometimes called sulphuretted hydrogen, will serve for an illustration. When this gas is breathed, the red corpuscles are darkened; and are not restored to their florid hue by oxygen, as they are when darkened by carbonic acid. When this gas and an oxyd of iron are in contact, double decomposition takes place, resulting in the formation of sulphide of iron and water. No one would expect iron in this state either to carry oxygen or to give it out in the capillaries. To the extent of this combination, the corpuscle permanently loses its functions, or in other words, dies.

Binoxyd of nitrogen, or nitric oxyd, is another highly poisonous gas. Like the preceding, it can not be inspired when undiluted, but mixed with atmospheric air, or other supporter of respiration, it may be inhaled in injurious quantities. This gas is characterized by an exceedingly strong affinity for oxygen. It is thus able to deoxydize many of the metals. When by inspiration it is brought in contact with the corpuscles, it can scarcely fail to deoxydize the iron, at least to a partial extent; and if it is able to reduce the sesquioxyd to a protoxyd, the blood is no longer arterial, and to the extent of this reduction, is unable to support the functions of organic life. By taking oxygen, this gas becomes an acid, and is, therefore, ready to unite with the oxyd of iron to form a salt. This is the gas most likely to contaminate nitrous oxyd, and is usually the cause of the darkening of the complexion, so often described in the books, and far too frequently seen. Nor does it await its arrival in the blood before it begins to undergo farther oxydation. In contact with free oxygen it is almost instantly changed to nitrous acid, and in the presence of watery vapor, almost as rapidly from nitrous to nitric acid. Much of it is thus changed to nitric acid in the air-cells of the lungs, and by its corrosive action on the mucous membrane, it renders it unfit for its special office, and the transfusion of gases through it does not take place with sufficient freedom. In this way the function of respiration is often badly damaged for days and weeks, after the use of impure nitrous oxyd. It is unfortunate that this poisonous gas is as likely to be obtained from pure as from impure nitrate of ammonia. For a more specific notice of this gas and its reactions, the reader is referred to Watt's Chemical Essays.

Defective respiration produces its deleterious results in various ways. If the supply of oxygen is so deficient in quantity that it fails to sesquioxydize the iron, of course the carbonic acid is not liberated; and if the iron, thus unchanged, passes to the left side of the heart, then blood, partially venous, will circulate in the arteries, and to the extent of this, the corpuscles are unable to carry the oxygen for the supply of force to the organic functions. And in this abnormal state, they seem to be equally unable to take up and retain the oxygen of reserve for the support of the voluntary functions. We forget, too, that although carbonic acid is the sole result of the complete oxydation of carbon, and water the ordinary result of the oxydation of hydrogen, yet, if the supply of oxygen be defective, many other compounds may result from the oxydation of these elements. In the body, as out of it, the partial oxydation of carbon forms carbonic oxyd, instead of carbonic acid. This oxyd, as is well known, is a deadly poison, when inspired. And when its *modus operandi* is clearly understood, we can readily recognize that, if formed in the blood, it is not less poisonous, than when it reaches it by inspiration. This gas is also characterized by a strong affinity for oxygen. If brought in contact with the red corpuscles in the arterial blood, it may take oxygen from the iron, reducing the sesquioxyd to the protoxyd, while by the oxygen thus received it is converted into carbonic acid, and will unite with the protoxyd to form carbonate of iron, and to the extent of this change, venous blood is formed in the arteries. The iron, having already given its half equivalent of oxygen to the carbonic oxyd, can not give it out in the capillaries, to supply vital force to the organic functions.

But instead of taking oxygen from the iron, or even while doing so, carbonic acid may combine with the oxygen of reserve, and to the extent of this, is the vital power of the voluntary functions lessened. This readily explains the sense of extreme prostration experienced in badly ventilated rooms, in prolonged paroxysms of asthma, or when, from any cause, the function of respiration is seriously defective.

If what is here written aids us in understanding better than before the important function of respiration, the object of the writer will be gained. To dentists the subject is a practical one. Our specialty originated practical anæsthesia, and to a great extent is guiding the investigations necessary for its further development. As general anæsthetics are administered by inhalation, correct ideas of respiration seem essential to their proper use. If apology were necessary, for not reading something more nearly related to every day practice, I would remind you that it would not be modest for me to discuss the details of a practice which has made so much progress since I abandoned it. Presuming that respiration is mainly carried on in the old way, I felt safer in selecting it, and have written this in obedience to your appointment.

